# LaueNN: neural-network-based *hkl* recognition of Laue spots and its application to polycrystalline materials

**DOI:** 10.1107/S1600576722004198

**Published:** 2022-06-15

**Authors:** Ravi Raj Purohit Purushottam Raj Purohit, Samuel Tardif, Olivier Castelnau, Joel Eymery, René Guinebretière, Odile Robach, Taylan Ors, Jean-Sébastien Micha

**Affiliations:** a Univ. Grenoble Alpes, CEA, IRIG, MEM, NRS, 17 rue des Martyrs, Grenoble 38000, France; b PIMM, Arts et Metiers Institute of Technology, CNRS, ENSAM, 151 boulevard de l’hopital, Paris 75013, France; c Université de Limoges, IRCER, UMR 7315, CNRS, Centre Européen de la Céramique, Limoges 87068, France; d Université de Haute-Alsace, IS2M, CNRS, UMR 7361, 3bis rue Alfred Werner, Mulhouse 68093, France; e Univ. Grenoble Alpes, UMR SYMMES CNRS-CEA, 17 avenue des Martyrs, Grenoble 38000, France; Ecole National Supérieure des Mines, Saint-Etienne, France

**Keywords:** synchrotron X-ray Laue microdiffraction, neural networks, *hkl* recognition

## Abstract

A feed-forward neural-network-based model is presented to index, in real time, the diffraction spots recorded during synchrotron X-ray Laue microdiffraction experiments. Data dimensionality reduction is applied to extract physical 1D features from the 2D X-ray diffraction Laue images, thereby making it possible to train a neural network on the fly for any crystal system. The capabilities of the LaueNN model are illustrated through three examples: a two-phase nano­structure, a textured high-symmetry specimen deformed *in situ* and a polycrystalline low-symmetry material. This work provides a novel way to efficiently index Laue spots in simple and complex recorded images in <1 s, thereby opening up avenues for the realization of real-time analysis of synchrotron Laue diffraction data.

## Abstract 2

An efficient neural network architecture to index Laue spots in synchrotron Laue microdiffraction patterns is presented. The efficiency and predictive capability of the model makes it applicable to real-time indexing of Laue microdiffraction data.

## 1. Introduction

Laue microdiffraction is an X-ray scattering technique for the determination of local microstructural parameters (strain and orientation) in materials (Tamura *et al.*, 2002 ▸; Chung & Ice, 1999 ▸). It is based on the recording and analysis of the ‘Laue pattern’ made from the digital image of the angular distribution of the scattering intensities on a 2D detector using a polychromatic incident X-ray beam. In materials science and Laue microdiffraction beamline setups worldwide [*e.g.* ALS (Tamura *et al.*, 2002 ▸), APS (Ice & Larson, 2000 ▸), ESRF (Ulrich *et al.*, 2011 ▸)], the Laue pattern from a single crystal is typically composed of a number of peaks ranging from ∼20 for a cubic unit cell to 500–1000 for a low-symmetry crystal. Each peak or ‘Laue spot’ is related to reflecting lattice planes defined by their Miller indices (*hkl*) [including harmonic (*nh nk nl*) planes where *n* is a positive integer]. The standard Laue microdiffraction experiment is a scanning X-ray scattering technique. It offers many experimental advantages when investigating bulk or microstructured specimens: high spatial resolution (from a few 100 nm to 1 µm), no sample rotation required to record the X-ray scattering signal (single-shot experiment), and high angular resolution on unit-cell shape parameters and crystal orientation. The standard data analysis has three main steps: peak search (image segmentation to a list of peaks), indexing (assigning Miller indices to peaks) and unit-cell parameter (or equivalently deviatoric strain) refinement (Barabash *et al.*, 2001 ▸; Plancher *et al.*, 2016 ▸; Zhang *et al.*, 2017 ▸; Petit *et al.*, 2015 ▸). For single-crystal Laue patterns and/or cubic structures, fast and reliable data analysis is available with several software packages. However, the Laue pattern complexity increases critically for low-symmetry crystals and/or when the X-ray-probed volume comprises several crystals, leading to superimposed patterns in a single image. Then, the data workflow is obstructed at the indexing step, which must segment the set of Laue spots into subsets, each corresponding to a single-crystal Laue pattern. The indexing of superimposed Laue patterns is thus of crucial importance for the reliability of the crystal orientation and lattice strains. In parallel, the production rate of data on synchrotron beamlines has increased several-fold during the past decade with increased photon flux of sources and very significant improvement of the detectors (speed, size, sensitivity). In a typical experiment performed at the French CRG-IF BM32 beamline at the European Synchrotron (ESRF) (Ulrich *et al.*, 2011 ▸), when investigating a polycrystalline material (depending on scattering power, grain size and level of disorder), anywhere from 3000 to almost 10 000 images of 8 MB can be collected per hour (sCMOS 2016 × 2018 pixels, 16 bit detector) during a sample raster scan, each image containing a superimposition of several single-crystal Laue patterns.

Several analysis tools have been developed by different groups dedicated to the interpretation of the Laue pattern originating from single-crystal (Dejoie & Tamura, 2020 ▸; Gupta & Agnew, 2009 ▸; Ouladdiaf *et al.*, 2006 ▸) and polycrystalline materials, for example, *XMAS* and by-products (Tamura, 2014 ▸; Kou *et al.*, 2018 ▸; Song *et al.*, 2019 ▸) and *LaueTools* (Robach & Micha, 2015 ▸). In these tools, after the spots have been precisely located on the detector frame, the indexing step relies on the comparison of the angle between two diffraction scattering vectors from two Laue spots, by means of a look-up table (LUT) calculated in advance for the crystal of interest. The LUT is built between fairly small numbers of *hkl*s to limit the computation requirements. In many cases, the indexing step is the most time consuming within the workflow. It can last several minutes (depending on the length of the LUT) using standard indexing parameters (number of tested pairs of spots, Laue pattern simulation and decision parameters from the analysis of some successfully indexed images) or even fail when dealing with low-symmetry structures such as polycrystalline monoclinic ZrO_2_ (Örs *et al.*, 2018 ▸). To address the speed-up related to the indexing step, other methods such as template matching and auto-encoder-based convolution neural network have been employed in recent years. Template matching involves comparing sections of experimental images with simulated images (Gonzalez *et al.*, 2004 ▸; Gupta & Agnew, 2009 ▸), whereas the auto-encoder-based neural network (NN) (Song *et al.*, 2019 ▸) relies on extracting features on a set of Laue images from a 2D raster scan and labelling them directly by skipping the indexing process. However, first the template matching or iterative classical indexing requires powerful parallel CPU/GPU clusters to index thousands of Laue patterns in a reasonable time. Also, such computational resources are often not readily available for researchers. Second, the labelling by Song *et al.* (2019 ▸) is not applicable to the analysis of a single image, even if it is a useful step to perform a partition of a set of Laue images for further standard indexing on a reduced number of images.

In this paper, we apply an NN-based model to the indexing of Laue images. Instead of intuitively applying the classical image-based convolution neural network (CNN), we employed a data reduction approach to convert the 2D Laue images to 1D angular distributions to enable a reliable classification. As a consequence, learning and predictive computing times are also reduced. The LaueNN model described in this paper has been optimized and tested to work with a maximum of 2000 experimental Laue spots per image. During the training, this model was able to achieve high training and validation accuracies in ∼25 s to ∼15 min for cubic and triclinic crystal systems, respectively, and it is able to recognize the *hkl* Laue indices of all spots in a Laue image, thereby allowing an efficient determination of orientation and unit-cell parameters of all the crystals probed. The model trained with synthetic data is based on the distribution of mutual angles between a Laue spot and its neighbouring spots in the Laue image. Details of the proposed method are given in Section 2. The validity and relevance of the model are then demonstrated through several case studies of experiments with increasing levels of complexity in Section 3.

## 2. Methodology

In this paper, the raw detector image is referred to as a Laue image, whereas Laue pattern refers to a set of Laue spots defining a single crystal. Thus in the case of a polycrystalline material, a Laue image is a superimposition of several Laue patterns of different diffracting crystals. This article will consider the Laue reflection geometry (detector on top of the sample, collection angles centred on 2θ = 90°) used by the BM32 beamline and other beamlines worldwide. This configuration will be employed for simulating Laue patterns that will form the basis of the training data set for the NN model, but it is straightforward to apply the same procedure to other geometries such as transmission.

### 2.1. Classical approach to indexing Laue patterns

Before discussing the proposed NN, in this section we present the classical method of indexing Laue patterns by peak-pair recognition (Heizmann & Baro, 1967 ▸; Riquet & Bonnet, 1979 ▸), which is implemented in most Laue indexing tools. This recognition works by finding several experimental peak-pair angles in the theoretical LUT of angular distances between *hkl* pairs. Prior knowledge of the unit-cell lattice parameters helps to restrict the tolerance angle to consider a valid recognition. We briefly explain the different elements of classical analysis here; interested readers will find more details in the literature (Petit *et al.*, 2015 ▸; Örs *et al.*, 2018 ▸).

**Figure 1:**
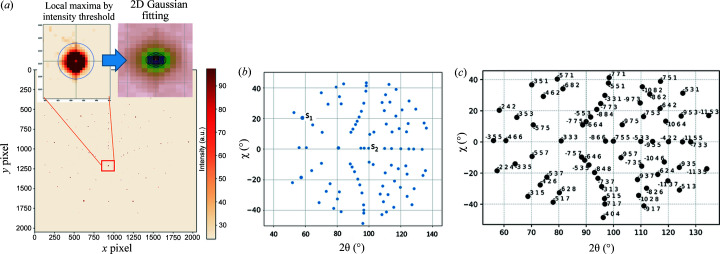
Classical single Laue diffraction pattern analysis steps. (*a*) Peak/spot detection by image processing in the detector space. (*b*) Laue spots represented in the angular space (scattering angles 2θ, χ); the mutual angle between a pair of spots is then compared with the theoretical LUT. (*c*) Result of the indexing (*hkl* Miller indices assigned to each spot). Illustrative example for a Ge single crystal.

The classical indexing in Laue diffraction consists of three main steps (illustrated in Fig. 1 ▸).

(1) The first step is to locate the diffraction peaks. It involves first resolving the local maxima in the recorded Laue image by image processing and then using these local maximum locations as initial guesses for the refinement of a 2D fit with a Gaussian peak shape model. Thus sub-pixel resolution can usually be achieved.

(2) In the second step, the spot positions are converted/projected in the angular space (two scattering angles 2θ, χ). The angular distance between an arbitrary pair of spots (*s*_1_, *s*_2_) is then compared with an extended LUT of the considered (unstrained) crystal structure within a given tolerance. From any matching pair of theoretical spots (*hkl*_1_, *hkl*_2_), two possible orientation matrices can be derived: 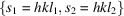 or 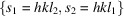 . Accordingly, two possible Laue patterns can be simulated and compared with the experimental one. A matching score 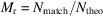 can be computed, where *N*_match_ is the number of simulated spots whose positions match the experimental spots within a given tolerance and *N*_theo_ is the total number of simulated spots. The accepted orientation matrix is the one with the highest matching score. Often multiple theoretical lattice plane pairs can be found in the LUT for a single selected pair of spots; for example, in a cubic system the angles between the normals of (111) and (100), (211) and (011), and (221) and (111) are all equal to 54.736°. Hence, due to crystal symmetry, the number of potential pairs-of-spots solutions can increase significantly with the tolerance angle of the LUT recognition. In order to ensure we find the highest *M*_r_, a large number of experimental spot pairs are tested following a screening approach of this trial and error brute-force method.

(3) In the final step, the retained orientation matrix is used as a guess to refine the crystal lattice parameters and crystal orientation. If lattice parameters for the unstrained lattice (*i.e.* strain free) are known, the deviatoric strain tensor components can be determined. Evidently, it is of highest importance to index unambiguously the spots belonging to the same crystal, otherwise the strain computation and crystal parameters will be prone to errors.

The classical indexing process works efficiently in the case of a high-symmetry crystal lattice and Laue images containing, in practice, only a few (<5) patterns coming from crystals probed by the X-ray beam simultaneously. To increase the indexing success, this procedure is nevertheless CPU intensive: the theoretical LUT is set to be sufficiently large, such that any two randomly chosen experimental spots will find their angular distances in the LUT. The time of computation is proportional to the number of trials of spot pairs which scales with 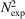 following a brute-force approach (where *N*_exp_ is the number of experimental spots found on the image). There are some workarounds to tackle having a large LUT and a large set of spots to be screened, such as considering only the high-intensity spots (whose *hkl* indices are likely to be small). However this becomes less evident and effective when the Laue image contains a few hundred spots, leading to ambiguously close spots belonging to a different crystal, which cannot be distinguished given the matching tolerance angle.

### 2.2. A neural-network-based approach to predict Laue spot *hkl*s

We propose here an alternative way to index the Laue pattern with improved speed and efficiency by employing a model based on a deep learning technique. Among the many other approaches available in deep learning, we used feed-forward neural networks (FFNNs) also referred as ‘multilayer perceptrons’ (MLPs). FFNN is a classification algorithm inspired by biological constructs and is a widely adapted network in different practical applications (Senthilkumar, 2010 ▸). The intention of this article is not to detail the FFNN model, which is reviewed elsewhere (Sazli, 2006 ▸); we report on the considerable speedup of this new procedure over the classical indexing technique for multi-grain and/or multi-phase Laue images recorded.

The FFNN is a type of family of NNs where the information only travels in one direction along the network, *i.e.* forward through multiple layers and finally to the output layer. There is no feedback from the output of the network back to the input. The reasons for choosing to work with reduced dimensionality data were twofold: firstly the training time of the NN should be kept as low as possible, thereby raising the possibility of training an NN for any material during the experiments; and secondly, the prediction time of an NN should be very fast, *i.e.* on the order of several hundred milliseconds, to be able to catch up with the detector read-out speed and index each Laue pattern in real time during an experiment. Another motivation was to be able to work with low-end standard computers (without a dedicated GPU), *i.e.* avoiding processing images (or 2D data) during training.

**Figure 2:**
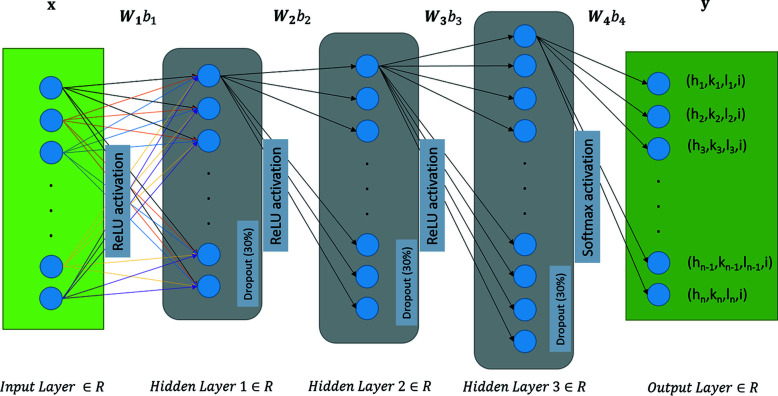
Generalized dense fully connected FFNN architecture for *hkl* indexing. The output class (*h*, *k*, *l*, *i*) refers to the *hkl* indices of crystallographic structure *i*. The *hkl* indices are reconstructed up to an index *n*. The parameter *i* is used to distinguish concomitant crystal structures.

The architecture of the FFNN model used in the current study is illustrated in Fig. 2 ▸. The input is the 1D vector **x** (no computations are performed at the input layer). The input vector **x** corresponds to a Laue descriptor/feature, corresponding to the angular distribution of peaks around a given reference peak (see details in Section 2.3). Three hidden layers of different neuron lengths are included between the input and output layers, each connected to the previous layer by their respective weight (**W**) and a bias constant (*b*). The choices pertaining to the number of neurons per layer and number of hidden layers are detailed in the next sub-section. Each hidden layer has an accompanying activation function and dropout ratio. The activation/transfer function defines whether a neuron should be activated or not, while dropout randomly deactivates a neuron during the training of the model. The rectified linear activation function (ReLU) was chosen to handle vanishing gradient for efficient learning and improved performance (Nair & Hinton, 2010 ▸), except in the final layer where softmax activation was used. A way of regularizing the model learning was to introduce dropouts between hidden layers (Srivastava *et al.*, 2014 ▸). Here a 30% dropout is introduced between each hidden layer. During the training process by back-propagation (Hecht-Nielsen, 1992 ▸), the weights in each of the neurons in these hidden layers are optimized to get the desired output class (**y**) for each **x** vector of input. In our case, the components (**y**) of the final output layer represent the probabilities of being the Laue indices *hkl*. In other words, it is the probability that the index of the reference peak chosen to build the input vector **x** is *hkl.* The number of output classes depends on the crystal symmetry considered. The output classes of several materials can be concatenated to allow the indexing of peaks in a multi-material environment (where a Laue image can be composed of contributions from more than one material). The number of outputs (Laue indices *hkl* to be recognized) can be multiplied by the number of distinct materials to be recognized (in Fig. 2 ▸ ‘*i*’ is used to distinguish the material).

In a general sense, one can write the output vector **y** as an activation/transfer function of the input vector **x** and various weights (**W**) and biases (*b*) of respective hidden layers:

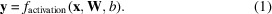

For each layer, one defines a single scalar bias *b* and weight vector **W** that has as many components as the number of neurons in that layer. The complete formulation of the NN presented in Fig. 2 ▸ is as follows:

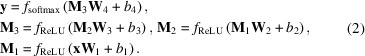


*f*_ReLU_ is the ReLU activation function which outputs the input only if the input is positive and formulated as follows:

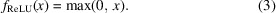


*f*_softmax_ is the softmax activation function which converts a vector of real numbers into a vector of probabilities (*i.e.* the sum of its coordinates is 1) and is formulated as follows:

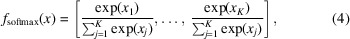

where *K* is the number of output classes (Laue indices *hkl*). The training of the NN is carried out by minimizing the cross-entropy loss function which is well suited for multi-classification purposes. The cross-entropy loss is the sum of the negative log-likelihood of probabilities and is defined as

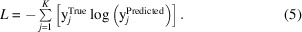




### 2.3. Meaningful feature for indexing Laue spots

The ability of an NN to learn efficiently depends strongly on the data features used. Just like for the classical indexing approach, our NN-based indexing relies on the recognition of mutual angular distances between Laue spots. We then used the mutual angular distances between spots as the input descriptor for the NN.

**Figure 3:**
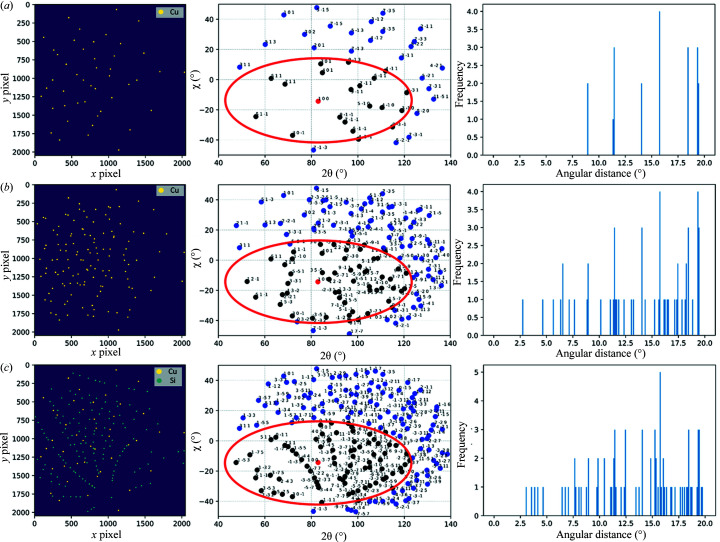
Simulated Laue image, ground truth indexing and angular distribution around a single peak for (*a*) a Cu single crystal, (*b*) Cu polycrystals (three crystals), and (*c*) Cu and Si single crystals. The leftmost subplots present the Laue spots in pixel coordinates, the middle plots present the Laue spots in scattering angle coordinates (the ellipse is a guide for eye to represent the spots within 20° of angular distance from the red spot), and the rightmost plots are the histograms of radial angular distributions (with fixed bins of 0.1°) of neighbouring spots around a single Laue spot.

Fig. 3 ▸ illustrates the process of generating 1D reduced data from the 2D data for the NN. For the training data set, we generate a large number of simulated Laue patterns from a uniform distribution of orientation. Then for each Laue pattern (each crystal orientation), the radial angular distribution around all simulated spots is computed to build the distribution for all Laue indices (*hkl*) found in the training set. This distribution is used as discriminative features for the NN.

For better recognition results, Laue images are simulated consisting of the union (superimposition) of several individual Laue patterns from randomly oriented crystals [Fig. 3 ▸(*b*)] and even of different crystal structures in the case of multiphase materials [Fig. 3 ▸(*c*)]. In Fig. 3 ▸, the angular distribution is shown for one selected spot; the 100 spot in Fig. 3 ▸(*a*) has only neighbouring spots of the same crystal, whereas in Fig. 3 ▸(*b*) the same 100 spot has additional neighbouring spots coming from the other two crystals, and Fig. 3 ▸(*c*) shows the angular distribution for the same 100 spot when another phase (single-crystal Si here) is present. The advantage of using a real experimental geometry for data set generation is also to capture the location dependency of the spots, *i.e.* the angular distribution varies significantly if the spot is in the centre or at the edges of the detector. Only spots lying within the detector bounds are accounted for in the histogram of angular distribution. Hence by simulating many random orientations, the NN is able to recognize the *hkl*s of spots even if they lie close to the detector edge (partial neighbouring).

In the NN framework, input vector **x** is the distribution of angular distance (with a bin size of 0.1°) for any given spot and the output/target neuron components of **y** will be the Laue indices *hkl*. Since each crystal structure is well defined by its space group, taking into account symmetries, Laue indices *hkl* in the output layer represent a crystal form made of the equivalent families of planes (*hkl*) whose indices are permuted or inversed according to symmetries. Let us illustrate this with copper. Copper belongs to the *Fm* 3 *m* space group, *i.e.* each *hkl* can be expressed by its 48 equivalent *hkl*s. That is, a copper 100 reflection is indistinguishable from 010, 001, 100, 010 and 001 along with their harmonics. This has to be taken into account during the generation of a training data set for the NN. In the case of the copper 100 reflection, all the equivalent *hkl*s and their harmonics are clubbed together and are represented by a single output neuron. Consequently, the 1D distribution of angular distance of the equivalent *hkl*s of (100) will be represented by a single neuron in the output layer.

### 2.4. Generation of training and validation data sets

To match real collected Laue patterns (finite recording exposure time, high-energy cut-off of incoming polychromatic beam), the synthetic Laue patterns have been partly modified and filtered. The Laue spot intensity scales mainly with the grain size, level of disorder and structure factor. An experimental Laue pattern can be composed of a fraction of all possible Laue spots, hereafter referred to as a ‘partial’ Laue pattern. We include the partial Laue pattern in the training data set by displacing the synthetic Laue spots following a normal distribution with σ = ±0.5 pixels, as well as removal of spots whose energies are larger than 20 keV to mimic real experimental conditions.

We were careful to generate a training data set without introducing bias by considering a uniform sampling of orientations when simulating the Laue patterns. We used the *Neper* code (Quey *et al.*, 2011 ▸) to generate a list of uniformly distributed orientations by taking into account the crystal symmetry (Quey *et al.*, 2018 ▸). For higher-symmetry structures, 500 orientations are often sufficient to describe the orientation space uniformly, whereas more orientations are required for low-symmetry structures (*e.g.* ∼2000 orientations for monoclinic symmetry). Of the total generated orientations, 20% are kept aside for the validation data of the NN. Care was also taken to avoid overlap between the training and validation data sets, as this could lead to strong overfitting.

Once the list of orientations is defined, the training and validation data sets of Laue images in single and polycrystalline configurations are generated by randomly picking the sampled orientations from their respective lists. From the generated Laue images, the 1D distribution of angular distances is constructed for each set of Laue indices *hkl*. Also the (synthetic) ground truth, *i.e.* the *hkl* indices for all spots in the validation data set, is stored to evaluate the performance of the model at the end of each training step.

### 2.5. Neural network architecture

The number of input and output neurons is fixed in the case of an FFNN architecture and there is no transfer of knowledge from the output to the input layer. In the current study, the NN model is constructed with the Keras (version 2.8; Chollet, 2015 ▸) backed by TensorFlow (version 2.8; Abadi *et al.*, 2015 ▸) Python libraries. The Adam (Kingma & Ba, 2014 ▸) optimization algorithm was used for the training. Grid search was performed to optimize the number of hidden layers, dropout ratio and number of neurons per hidden layer required to efficiently address the complexity of indexing. Details of the Python notebook scripts corresponding to the grid search optimization are provided in the supporting information. The number of hidden layers was varied between one and ten, and it was observed that one hidden layer was not sufficient to obtain the desired accuracy, while anything more than three hidden layers gave no visible improvement and increased the risk of overfitting and the training and prediction times by a factor of 2. Similarly a dropout ratio of 30% with the following number of neurons per layer was found to be satisfactory: Hidden layer 1 has the same number of neurons as input **x**. The number of neurons is 7 times the number of neurons in input **x** for hidden layer 2 and 15 times for hidden layer 3. And for the output layer, the number of neurons depends on the number of Laue indices *hkl* found in the training data set.

Owing to crystal symmetries, some *hkl*s appear more often than others in the Laue image, leading to an imbalance and possible overfitting. Here we use a penalty associated with the frequency of having an *hkl* within the training data set which is introduced in the cross-entropy loss function [equation (5)].

**Figure 4:**
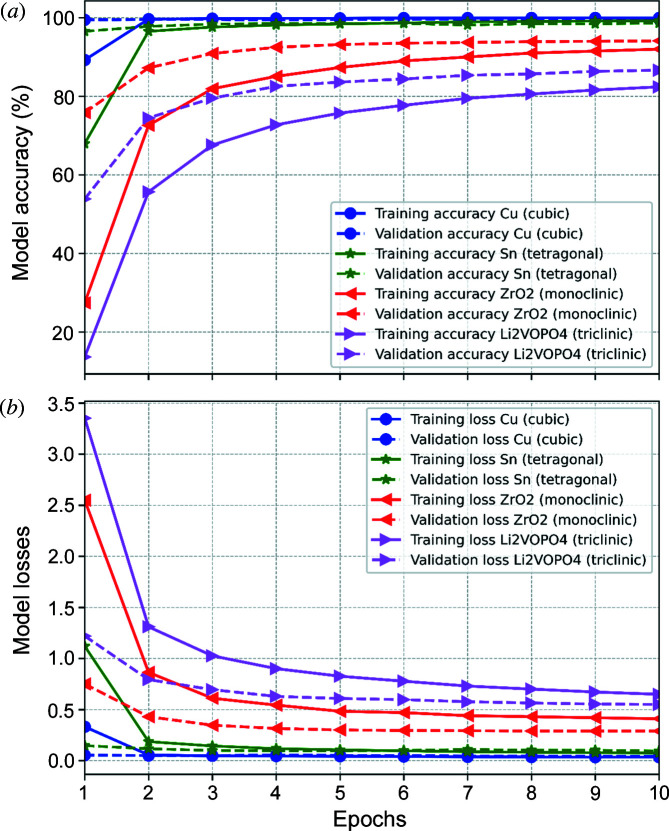
(*a*) Training and validation accuracy; (*b*) losses for *hkl* recognition in different crystal systems

Fig. 4 ▸ shows the training and validation accuracies [Fig. 4 ▸(*a*)] and losses [Fig. 4 ▸(*b*)] for different crystal symmetries. Accuracy is a straightforward metric for model performance. Accuracy is computed at the end of each epoch. An epoch here refers to one complete cycle of the training data set passing through the network. At the end of each epoch, the training accuracy is calculated as the ratio of total good predictions over the total number of predictions on the training data set, and the validation accuracy is similarly calculated over the validation data set. The training loss is calculated and updated as each sample passes through the network. The loss function [equation (5)] is minimized by updating the weights of the neurons in the NN during the learning. At the end of each epoch both training and validation losses are calculated on the training and validation data sets, respectively. For cubic, hexagonal (not shown here) and tetragonal crystal systems, validation accuracies >95% were reached quickly, whereas for monoclinic and triclinic systems, validation accuracies close to 90% were recorded. For lower-symmetry crystals (more spots are present per Laue pattern), the architecture can be further optimized and higher accuracies should be achievable. However, validation accuracies of ∼90% are still very good for our purpose.

All training and predictions were carried out on a standard 8 core (2.40 GHz) laptop. Table 1 ▸ presents the figures of the NN training. Training a model for higher-symmetry crystals takes seconds to minutes, whereas for a triclinic system it takes approximately 20 min. The prediction time for all spots in a Laue image for all crystal systems is <1 s.

To evaluate the trained model performance on synthetic data, multiple random simulation runs (1000) were performed with single-crystal/polycrystalline copper simulated Laue images. The statistics of these runs are presented in Table 2 ▸. Note that the NN training data set included only Laue images comprising one to a maximum of five crystals and yet the NN manages to predict the *hkl*s in a Laue image comprising ten crystals with >90% accuracy. We also observed that the time for prediction increased with the number of spots per Laue image. This can be reduced by employing multiprocessors during prediction, thus making the on-the-fly indexing of Laue patterns a reality.

Here we summarize the procedure of LaueNN before application to experimental cases.

(i) To simplify and accelerate the training and prediction of Laue patterns, as a first step we provide a data reduction approach to convert the 2D detector images (2000 × 2000 pixels) from pixel coordinates to a more meaningful 1D angular distribution descriptor. As a result, the NN model requires less processing power.

(ii) Often NNs require large data sets with manual labelling of data by experts. By using purely simulated data taking advantage of crystallographic symmetries we avoid any bias or mistakes during the generation of training data sets. Instead of training data sets of a few hundreds of thousands of samples, we reach validation accuracies of >95% with only a few thousand samples. The procedure to build the training and validation data sets for all crystal systems is provided.

(iii) A single NN architecture has been optimized to work for all crystal systems. Generation of training data to train the NN model to higher accuracies takes <20 min and can be done at the start of data collection experiments or beforehand at the user laboratory or the beamline. The Python notebook scripts that describe the various steps from the generation of the training data set to predicting Laue spots are provided in the supporting information.

(iv) The NN model provides the prediction of the *hkl*s of spots and not the orientation itself. Prediction of all *hkl*s in a single Laue image takes <1 s. Selected pairs of recognized *hkl*s are tested to propose an orientation matrix. The proposals are validated and sorted via the matching rate *M*_r_ defined in Section 2.1.

(v) The above-mentioned methodology is optimized to enable real-time indexing of Laue patterns during experiments, thereby making it possible to locate interesting features in specimens and adapt experiments during beam time.

## 3. Application to experimental data

So far we have demonstrated the validation of the NN on simulated Laue patterns. In the following we present the NN performance obtained as an experimental Laue image data set collected on the BM32 beamline. The configuration of the experimental setup is illustrated and explained by Ulrich *et al.* (2011 ▸) and Örs *et al.* (2018 ▸): top reflection geometry, sample tilted by 40°, 5–22 keV energy range of the incoming beam, and ∼70 mm detector-to-sample distance. Three illustrative cases will be shown: a two-phase material (microcrystal on a substrate), a polycrystalline textured metallic alloy (cubic lattice) deformed *in situ*, and a polycrystalline material with sub-micrometre-sized crystals and a low-symmetry unit cell.

### 3.1. GaN nanowires on an Si substrate

**Figure 5:**
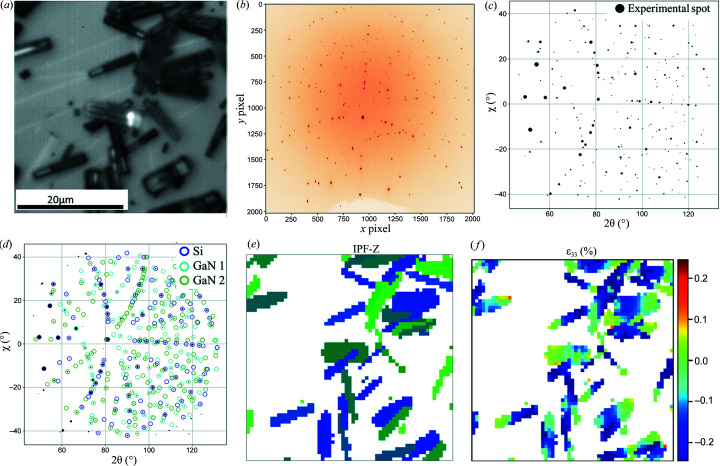
Laue diffraction on GaN embedded in an Si substrate. (*a*) Optical microscopy image of the scanned region (the white spot corresponds to the beam position), (*b*) recorded Laue image from the scanned region centre, (*c*) Laue image in scattering angle space (the spot size is proportional to the intensity), (*d*) NN indexing and classification of phases in a Laue image, (*e*) reconstructed IPF-Z map of highest matching score for GaN crystal in the scan region, and (*f*) ɛ_33_ (*c* axis of GaN) strain component.

This first illustrative example consists of GaN nanowires deposited on an Si substrate and demonstrates the ability of LaueNN to detect several crystallographic phases in a single Laue image measurement. The wires have been designed for UV emission and are grown by metal–organic vapour phase epitaxy on *c*-sapphire substrate using a silane-assisted method (Koester *et al.*, 2010 ▸, 2011 ▸). The experimental details related to growth conditions and structural characterization are given by Grenier *et al.* (2021 ▸). The diameter of the wires is about 1 µm and the length is 13 µm. An important feature is that they are grown in two parts as shown by the contrast in the optical microscopy view in Fig. 5 ▸(*a*). The bottom, in white contrast, is heavily doped with silane and has a selective layer coating that suppresses lateral growth. In the top part [in dark contrast in Fig. 5 ▸(*a*)], a quite complex stacking optimized for strain management and UV emission is grown laterally, *i.e.* a GaN spacer, two AlGaN gradients increasing the Al content from 0 to 30% then to 60%, and then the active emitting region made of 5 × (2.5 nm GaN/5 nm Al_60%_Ga_40%_N) quantum wells. This core–shell growth occurs on non-polar *m*-plane {1010} wire sidewalls and is associated with a non-regular hexagonal section. The long direction is oriented along the *c*-axis direction of the GaN hexagonal crystal. These nanowires were mechanically dispersed on a silicon (001) substrate. When they do not interact with each other (stacking, electrostatics…), the *m*-plane sidewall facet of the wires is parallel to the (001) silicon surface.

The whole area of Fig. 5(*a*)  ▸ (41 × 41 µm) was scanned with the beam (beam size *V* × *H* was 300 × 700 nm). Since the X-ray beam can illuminate the Si substrate through the nanowires, the Laue image always has spots coming from the Si substrate, and possibly from the GaN phase. At some places a few nanowires overlap on top of each other, thus producing superimposed Laue patterns. Using the procedure explained before, a single NN model was trained with a combination of one crystal of Si and a maximum of three GaN crystals. The training data set consisted of the following combination (500 uniform orientations were sampled for Si, while 1000 uniform orientations were sampled for GaN): single-crystal patterns of Si in the absence of GaN, a single crystal of GaN with a single crystal of Si, two crystals of GaN with a single crystal of Si and three crystals of GaN with a single crystal of Si. The training took 15 min and the resulting model had a validation accuracy >95%. Fig. 5 ▸(*a*) shows the Laue scan region of the microstructure taken with the beamline optical microscope, Fig. 5 ▸(*b*) shows the Laue image (in pixel coordinates) for one of the measurements coming from the centre of the scan region, Fig. 5 ▸(*c*) shows the Laue spots in scattering angle space and Fig. 5 ▸(*d*) shows the identification of the crystal orientation from the NN.

Indexing was performed by means of the LaueNN method; following the procedure of Section 2.2, the matching score of all possible orientation matrices is evaluated from all the *hkl* predictions. The indexing procedure was repeated for all Laue images recorded during a sample raster scan. The NN was able to identify up to three distinct Laue patterns (successfully separating the spots belonging to the Si and the GaN crystals) from experimental images. Fig. 5 ▸(*e*) shows the reconstructed map of the highest matching score for a GaN crystal per Laue image in inverse pole figure (IPF) colour (along the *z* axis; normal to the substrate surface) for hexagonal symmetry.

The identified orientations are coherent with a very good matching score for both GaN (*c* axis lying in the surface plane) and Si(001). Once the diffraction spots of each crystal in each Laue image are identified with good confidence, the next step is to refine the lattice parameters of the crystals or equivalently the components of the deviatoric strain with respect to the reference lattice parameters (for GaN the following reference lattice parameters were used: *a* = 3.189, *b* = 3.189, *c* = 5.185 Å, α = 90, β = 90, γ = 120°). As expected, the strain in the Si wafer (not shown here) is negligible (on the order of 10^−5^), whereas for GaN a gradient of strain is observed along the nanowire axis [as shown with the map of longitudinal strain ɛ_33_, Fig. 5 ▸(*f*)]. The ɛ_33_ strain evaluated from the identified orientation presents a bi-modal distribution, which is a consequence of the way the nanowires were grown, as mentioned before. The strain resolution is estimated at 0.01%. The heavily doped silane regions (white contrast in the optical image) are unstrained along the *c* axis as expected, and the dark contrast region of the nanowire presents a clear compressive (*i.e.* negative) strain level of about 0.2%. This demonstrates that LaueNN can work directly with multi-phase Laue images and neither post- nor pre-processing of data to handle the Si peaks is necessary anymore (as was done previously with the classical indexing scheme).

Here, the duration for data acquisition was about 1 h (61 × 61 Laue images, 1 s exposure per image). The total time to extract Laue spots from the images, predict and construct three orientation matrices, and refine strains (five iterative step strain refinement) with an 8 CPU standard laptop was about 30 min (0.5 s per image). The individual time of each sub-process on a single CPU was as follows: extraction of Laue spots from the images (∼0.4 s), prediction of *hkl* (∼0.4 s), construction of the orientation matrix (cycling through high prediction accuracy spots ∼1.5 s), strain refinement (∼0.2 to 1.5 s depending on the number of strain refinement steps).

### 3.2. *In situ* deformation of high-symmetry polycrystals

**Figure 6:**
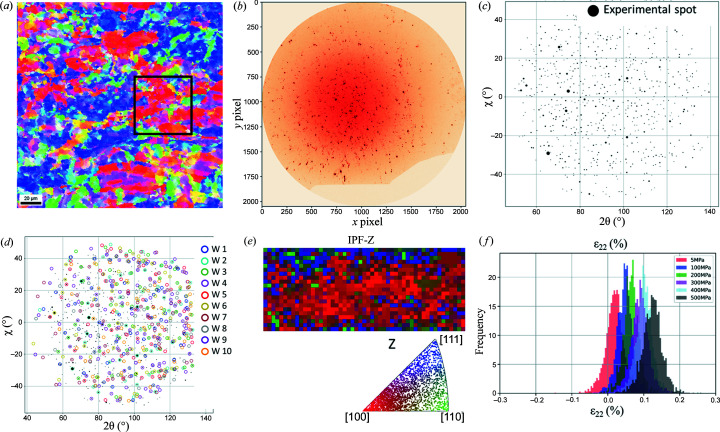
(*a*) EBSD IPF-Z map of tungsten, with a black box marking the approximate area scanned during Laue diffraction; (*b*) recorded Laue image from the scan region centre; (*c*) Laue image in scattering angle space (the spot size is proportional to the intensity); (*d*) NN indexing of polycrystals in a Laue image (ten crystals of W are indexed); (*e*) reconstructed IPF-Z map (pixel size: 1 × 2.5 µm) of the highest matching score for a W crystal in the scanned region along with all the indexed orientations presented in an IPF; (*f*) principal deviatoric ɛ_22_ (tensile direction) strain component.

This case study demonstrates the efficiency of the LaueNN model for fine-grain polycrystalline material exhibiting a crystallographic texture. We used a polycrystalline sample of tungsten (W) deformed *in situ* at room temperature with a dedicated tensile rig mounted on the Laue microdiffraction setup at BM32. The reason for investigating W is twofold. First, the yield stress for tungsten is large so that the specimen can be loaded *in situ* to high stress without initiating plastic strain. Second, the elastic behaviour of W crystals is isotropic, so that there is no spatial discontinuity in the mechanical behaviour between different grains. As a result, a tensile test should add a uniform stress field in the specimen, whatever its microstructure. According to the electron backscatter diffraction (EBSD) analysis [Fig. 6 ▸(*a*)], the crystallographic texture of the specimen shows two main components: 100 and 111. The microstructure constitutes large clusters of grains with similar orientations, either 100 (blue on the figure) or 111 (red). The average grain size is 5 µm. We used an earlier setup of BM32 where the beam size was 1.4 × 3.5 µm (*V* × *H*). Even if the penetration of the X-ray beam into W is small for the energy range of the incident beam (<10 µm), we observed that each Laue image [Fig. 6 ▸(*b*)] contains the superimposition of about ten Laue patterns, indicating a complex substructure of each grain made of a number of small crystals slightly misoriented from each other. The specimen was loaded *in situ* under uniaxial tension, from 5 to 500 MPa in steps of 100 MPa, and raster scans of 50 × 50 µm with a 1 µm step along *x* and a 2.5 µm step along *y* were carried out with the X-ray beam. We used markers on the specimen surface to consistently scan the same region of interest (ROI) during the tensile test (1 µm precision on the ROI position). The scan ROI mostly comprises grains with a 100 orientation.

Here the LaueNN model was trained for tungsten material with a combination of up to ten crystals per Laue image, knowing that experimentally we may have sometimes more than ten crystals per Laue image. The training took 15 min in total and the resulting model had a validation accuracy >95%. As shown in Fig. 6 ▸(*c*), the number of experimental spots per Laue image is around 700 (a single crystal of tungsten creates around 60 spots for the experimental setup and the energy band used). The model comfortably indexes ten tungsten crystal as shown in Fig. 6 ▸(*d*). Fig. 6 ▸(*e*) shows the reconstructed map of the highest matching score for a tungsten crystal per Laue image in inverse pole figure (IPF) colour (along the *z* axis; normal to the specimen surface) for cubic symmetry. To avoid uncertainties in the strain calculations, only the top five highest matching score crystals are kept per Laue image. This is based on the observation that the top five highest matching score indexed results are very likely from the crystals close to the surface and not too deep in the probed volume in order to reliably assess strain. The five indexed crystal per Laue image are provided in the accompanying IPF in Fig. 6 ▸(*e*), which suggests a strong (100) orientation cluster, in good agreement with the EBSD analysis.

Fig. 6 ▸(*f*) shows the distribution of the deviatoric elastic strain (ɛ_22_) along the tensile direction in the scanned zone for six applied stress levels. The mean of the local strains (other principal components not shown here) is in good agreement with the applied macroscopic stress levels (Young’s modulus of tungsten is around 400 GPa). As a result of the elastic isotropy of the W crystal, one could have expected a uniform stress field at each loading level, which is not the case. Here, the width of the strain distribution [shown in Fig. 6 ▸(*f*)] is much larger than the accuracy of the Laue technique, which is better than 10^−4^ (Plancher *et al.*, 2016 ▸; Petit *et al.*, 2015 ▸). The observed stress distribution has to be related to the manufacturing process of the W plate used to machine the specimen, involving complex thermal treatments and plastic deformation, known to be at the origin of a complex field of residual stresses at the intragranular scale.

This case demonstrates the ability of the LaueNN model to predict *hkl*s of spots in complex Laue images of polycrystalline materials for further strain refinement. Individual Laue images contained more than 700 spots, coming from at least ten crystals per scan point. The indexing and strain refinement of five crystals per image with an 8 CPU standard laptop took 125 min for 1071 images, *i.e.* ∼7 s per image (prediction of *hkl* indices for all spots for each Laue image averages at 0.8 s per image).

### 3.3. Strains in low-symmetry polycrystals

This case study demonstrates the capability of the LaueNN model to resolve complex Laue images acquired on low-symmetry monoclinic zirconia polycrystalline material that has undergone the solid-state martensitic phase transition (from tetragonal to monoclinic) and thus exhibits a very subdivided and complex microstructure with microcracks (Ors *et al.*, 2021 ▸; Guinebretière *et al.*, 2022 ▸). Low-symmetry crystal Laue patterns are often difficult to index using the conventional approach described in Section 2.1, as screening of a large number of spots is necessary to ensure a result. This can take several minutes to index a single Laue pattern. With the images considered here from a polycrystal, the standard procedure often did not succeed with a set of parameters for several tens of minutes. An indexing based on prior knowledge of the orientation needed to be developed (Örs *et al.*, 2018 ▸), wherein the orientation information coming from EBSD mapping performed *ex situ* on the same area was used as an initial guess to index the Laue patterns of monoclinic zirconia. This reduced the indexing time from hours to ∼15 min per image. Here we demonstrate that, by employing the LaueNN model, the indexing time can be reduced to <10 s per image for zirconia, without requiring any additional prior information. Each experimental Laue image for zirconia consists of 700–1000 spots, corresponding to several crystals of sub-micrometre size diffracting in the X-ray-probed volume. The samples were probed with a beam size of 300 × 300 nm. However, many of the diffraction spots formed ‘partial’ Laue patterns (see definition in Section 2.4); a full Laue pattern of a monoclinic zirconia single crystal should contain around 700 spots.

**Figure 7:**
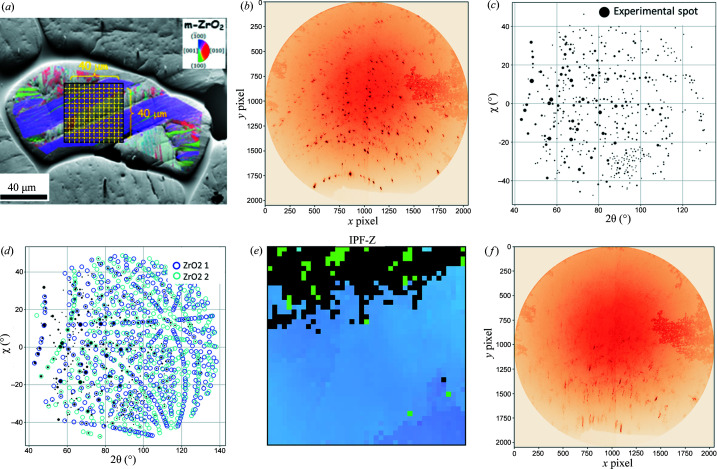
(*a*) EBSD map of the monoclinic zirconia polycrystal [taken from Fig. 4 ▸ of Ors *et al.* (2021 ▸) with permission from Elsevier], (*b*) recorded Laue image from the scan region centre, (*c*) Laue image in scattering angle space (the spot size is proportional to the intensity), (*d*) NN indexing of polycrystals in a Laue image (two crystals of ZrO_2_ are indexed), (*e*) reconstructed IPF-Z map of the highest matching score for a ZrO_2_ crystal in the scan region (black pixels are non-indexed Laue images), and (*f*) typical Laue image in the non-indexed region.

The model was trained with a data set consisting of up to five crystals per Laue image. Due to the low symmetry of the crystal, 2000 orientations were sampled uniformly for the training data set. The NN took 25 min to reach validation accuracies of >95%. Fig. 7 ▸(*a*) shows the EBSD map of the region probed by X-rays. Fig. 7 ▸(*b*) shows the Laue image of a single measurement coming from the centre of the scanned region (the cloudy pattern on the right side of the detector image is an artefact on the detector as a result of humidity). Fig. 7 ▸(*c*) shows the Laue image in scattering angle space, multiple partial Laue patterns are present and the model comfortably indexes two crystals of ZrO_2_ with a good matching score [Fig. 7 ▸(*d*)]. The remaining non-indexed spots are few and it is difficult to associate them with any orientation with good confidence. Fig. 6 ▸(*e*) presents the IPF-Z (*Z* being normal to the specimen surface) map of the highest matching score for the zirconia crystal per Laue image. The EBSD image in Fig. 6 ▸(*a*) was generated using the *Channel5* software from HKL Technology (HKL colour code), while Fig. 6 ▸(*e*) was plotted with the open-source *MTEX* software (Bachmann *et al.*, 2010 ▸), hence explaining the difference in colours. However the orientations are similar. The non-indexed data present a complicated Laue image with faint streaks and no proper spots [Fig. 6 ▸(*f*)]. Since the model was never trained with streaking or large displacement of spots, it fails to index such cases. Note that for the classical method the streaks present similar difficulty during indexing.

**Figure 8:**
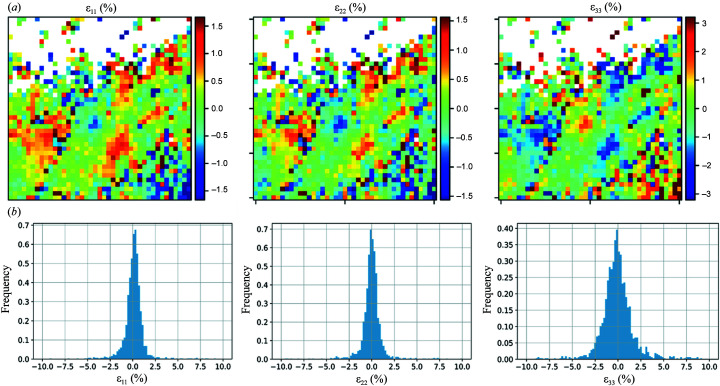
Principal deviatoric strain in the crystal reference frame for zirconia: (*a*) strain distribution for the highest matching score crystal per Laue image and (*b*) strain distribution for all the indexed grains.

Strain refinements were carried out for the indexed grains, and the distributions of the principal deviatoric strain components are presented in Fig. 8 ▸. The resulting distribution is very much coherent with the distribution obtained previously using the EBSD plus conventional approach [refer to Fig. 4 of Ors *et al.* (2021 ▸)]. The very heterogeneous distribution of strains hints at the presence of micro-cracks as a result of the solid-state phase transition experienced by zirconia, revealing huge internal stresses in the gigapascal range.

This case demonstrates the ability of the proposed LaueNN model to predict *hkl*s in complex polycrystalline low-symmetry material. The indexing of two crystals of zirconia per image with an 8 CPU standard laptop took 30 min for 1681 images (*i.e.* ∼1 s per image).

## 4. Discussion

As mentioned before, the objective of this work was to introduce a methodology to speed up the indexing of Laue patterns reliably. A feed-forward-based NN, LaueNN, is successfully employed for the prediction of Laue spots. The LaueNN method is available as an open-source GUI under the name *LauetoolsNN* on the PYPI repository (https://pypi.org/project/lauetoolsnn/). One could also employ other NN architectures such as the CNN model (LeCun *et al.*, 2015 ▸) for the problem of indexing. A CNN-based autoencoder has been proposed recently at the Advanced Light Source beamline (Song *et al.*, 2019 ▸) to extract features from Laue diffraction data to aid in the process of indexing. CNN models work directly on the 2D images and can be both memory and CPU demanding. The advantage of CNN models is that they extract features automatically and then classify them. However, the 2D nature of the Laue pattern signal is only apparent for the purpose of orientation recognition. It comes from the particular geometrical projection of 3D reciprocal space directions (from the origin to reciprocal space nodes) onto a detector. Due to the limited range of measurements (energy range, finite size of the detector), there is only a partial perspective of the crystal structure and its orientation. Moreover, experimental Laue images made of superimposed Laue patterns prevent CNN models from an efficient detection of all individual Laue patterns. Consequently, instead of using a CNN to extract features, we identified the main feature that affects the indexing of Laue patterns, *i.e.* angular distribution between spots. By employing this data reduction, we keep only the relevant features/variables that are provided to a fully connected dense NN. This helps us to design and train an NN for any material in very short time with just synthetic data. Similarly by employing a simpler NN architecture the time taken by the NN to predict the *hkl*s of spots is also reduced. Note that one can employ the 1D CNN-based model for similar problems by employing fewer trainable filters compared with the neurons in the MLP model employed here. In this work, importance was given to a network that is less memory intensive and quicker in terms of both training and prediction, hence the choice of MLP over 1D CNN. However, the advantages of CNN-based models are significant and both 1D and 2D CNN-based models will be tested in future work, for example, to identify lattice defects such as geometrically necessary dislocations from the 2D peak shapes of the Laue pattern. Apart from the machine learning models, several other approaches such as Hough transform (Wenk *et al.*, 1997 ▸) to index the Laue zone axis (similar to EBSD indexing), template matching (Gupta & Agnew, 2009 ▸) or pattern matching (Dejoie & Tamura, 2020 ▸) have been applied successfully for the indexing of Laue patterns. However their efficiency reduces as soon as multiple grains are probed simultaneously, leading to complicated superimposed Laue patterns. For example, template matching is very efficient for single-crystal indexing, but as soon as polycrystals are measured in the Laue image, the accuracy drops below 80%. This article provides an easier and better way to index more than ten crystals in a Laue image at good speed without compromising the accuracy. Even though the LaueNN model can index spots in <1 s, finding the crystal orientation can add up to several seconds to the total process of indexing. The total time per Laue image can be reduced to 1 s per image by employing parallel jobs. The model can be coupled with Hough transform or node/graph methods to further speed up the indexing process.

*In situ* or *operando* studies where any structural (phase transition, thermal expansion *etc*.) or microstructural (appearance of defects, strain relaxation *etc*.) evolution takes place can really benefit from the present model, with which real-time indexing is possible. The LaueNN model provides validation accuracy >95% for Laue images comprising up to 2000 spots. For materials science applications, the number of spots in Laue images is often well within 2000. For low-symmetry crystal systems such as triclinic, higher validation accuracy (*i.e.* >95%) could be reached by increasing the number of hidden layers. The input feature of angular distribution for the model can be further tuned depending on the Laue image. For example, in the current study we used a fixed bin width of 0.1° with the mutual angles of spots within a 20° radius when building the angular distributions. The choice of bin width also influences the results: very well defined peaks in the Laue image can be detected with sub-pixel resolution (0.1°). In addition, a much larger radius (≫20°) can be used to capture the distinct angular distribution of a spot of interest with respect to its neighbours. The model can be further tuned according to the complexity of the Laue images to achieve the desired accuracy of the *hkl* predictions.

The ability of the NN to successfully index the *hkl*s of spots depends strongly on the peak-search process (step 1 of the data workflow). This works well in the case where the Laue images are composed of nice round and isolated peaks/spots. But in some cases, due to crystalline defects and strain, the shapes of the diffraction spots in the Laue image are far from perfect circles, which can be seen in the zirconia Laue image in Fig. 7 ▸(*f*). In the case of streaks or elongated peaks, the peak search looks for the barycentre of the 2D peak. Currently in the training data set, data augmentation is carried out only by displacing peaks at the sub-pixel level. One way to include elongated peaks would be to include a strained simulated data set in the training data for better indexing.

The input for the NN can also include additional data such as intensities (namely based on structure factors but also models taking into account the changing absorption of X-rays in the materials with respect to Laue spot energy) or roundness/profile of the spot to extract more information and not just their *hkl.* Further research is underway to develop multiple neural-network-based models to create an automated pipeline for Laue data treatment and analysis.

## 5. Conclusions

In this paper we have proposed LaueNN, an NN model for the *hkl* indexing of Laue spots recorded on polycrystalline materials, which works even for crystals belonging to low-symmetry space groups. This was done with dimensionality reduction of data from a 2D Laue image to a 1D radial angular distribution of spots. An FFNN model was then used to learn from these 1D angular distributions to recognize *hkl*s of spots in Laue images. By employing synthetic data (subsequently avoiding any bias on ground truth labelling), the whole process of training and indexing was made free of any user intervention. The ability of this NN to index superimposed Laue patterns with very high speed and good accuracy was demonstrated with three experimental case studies of varying complexities. We demonstrated that the model can learn very well from multi-phase superimposed Laue patterns and can index and classify them to the proper phase. The proposed approach supersedes the conventional brute-force indexing method based on screening the recognition of the mutual angle of two spots. In particular, the analysis time for the Laue image with multiple crystals from a low-symmetry crystal is reduced down to ∼1 s.

Real-time/on-the-fly indexing of synchrotron polycrystalline or complex superimposed Laue patterns is now possible with the proposed LaueNN model, thereby providing users with valuable and rapid feedback during data collection on the beamline and accelerating the data treatment from raw data to reliable and interpretable structural parameters. The following model has already been implemented at the BM32 beamline, ESRF. Real-time indexing of several users’ experiments on the BM32 beamline has already been successful. In addition, predictions can be made offline in standard laptop multiprocessors in user laboratories. Future improvements are foreseen by combining the reported single-image analysis with new methods to treat a data set of images based on the relationships between images to index a set of Laue patterns (from a raster scan or *in situ* monitoring) by means of machine learning tools.

The data for the results presented in the article as well as the NN model and complete GUI source code can be accessed via the PYPI repository using pip install lauetoolsnn or via https://github.com/ravipurohit1991/lauetoolsnn. Additional information regarding the reproducibility of the results presented in the article and example Jupyter notebooks are provided in the supporting information.

## Supplementary Material

Supporting comments and results of the neural network. DOI: 10.1107/S1600576722004198/nb5322sup1.pdf

## Figures and Tables

**Table 1:** Neural network training statistics of different polycrystalline (up to five crystals) crystal systems Prediction time for one Laue image is also provided.

Compound	Crystal system	Maximum number of spots per Laue image	Model training time (s)	Total trainable parameters	Validation accuracy of the model (%)	Time to predict *hkl*s in one Laue image (s)
Cu	Cubic	225 ± 5	25	2711518	99.7	0.25
Ti	Hexagonal	525 ± 7	180	10304228	98.2	0.33
Sn	Tetragonal	806 ± 8	180	10304228	98.6	0.40
ZrO_2_	Monoclinic	2060 ± 27	750	59130894	94.4	0.65
Li_2_VOPO_4_	Triclinic	5200 ± 25	1200	182766262	88.2	0.86

**Table 2:** Statistics of model performance on 1000 random simulation runs for Cu

Number of crystals simulated	Total spots per Laue image	Prediction time per Laue image (s)	Accuracy of prediction (%)
1	45 ± 2	0.23 ± 0.015	100
2	90 ± 3	0.42 ± 0.025	99.99 ± 0.008
3	135 ± 3	0.62 ± 0.022	99.99 ± 0.01
4	180 ± 5	0.82 ± 0.028	99.87 ± 0.2
5	225 ± 5	1.00 ± 0.036	99.64 ± 0.4
6	270 ± 5	1.21 ± 0.036	99.09 ± 0.5
7	315 ± 6	1.42 ± 0.039	98.27 ± 0.7
8	360 ± 6	1.62 ± 0.047	96.71 ± 1.0
9	404 ± 6	1.82 ± 0.055	94.60 ± 1.25
10	450 ± 7	2.01 ± 0.049	92.26 ± 1.34
