# Supplementary material for: LaueNN: neural-network-based hkl recognition of Laue spots and its application to polycrystalline materials
Source: J Appl Crystallogr. 2022 Jun 15;55(Pt 4):737–50. doi: 10.1107/S1600576722004198 (PMC9348891; doi:10.1107/S1600576722004198)
Supplement: Supplementary file 1 [file j-55-00737-sup1.pdf]

JOURNAL OF APPLIED CRYSTALLOGRAPHY

Volume 55 (2022)

Supporting information for article:

LaueNN: Neural network based *hkl* recognition of Laue spots and its application to polycrystalline materials

Ravi Raj Purohit Purushottam Raj Purohit, Samuel Tardif, Olivier Castelnau, Joel Eymery, Rene Guinebretiere, Odile Robach, Taylan Ors and Jean-Sebastien Micha

1. Python notebook scripts

In the github repository of the project example notebook scripts are included to build a neural network model for any crystal symmetry as described in this article. These notebook scripts aims to provide a general tutorial into the complete flow of LaueNN method.

Step 1: Generation of training dataset script: https://github.com/ravipurohit1991/lauetoolsnn/blob/main/lauetoolsnn/example_notebook_scripts/Step1_Generation_dataset_LaueNN.ipynb

This notebook script provides information on how the output *hkl* class of the neural network is build given any crystallographic symmetry. Once defined, the simulated Laue patterns training dataset is generated and saved.

Step 2: Training a neural network: https://github.com/ravipurohit1991/lauetoolsnn/blob/main/lauetoolsnn/example_notebook_scripts/Step2_Training_LaueNN.ipynb

This notebook give introduction to defining a neural network architecture. Since the input and output classes are already generated, it is possible to define another neural network architecture, for example a 1D CNN model that takes in same input and output. Additionally another script is provided that helps the users to play with different hyper parameters of the model architecture to optimize it specifically for their case (Sub-step 2a: Optimize hyper parameters of neural network architecture: https://github.com/ravipurohit1991/lauetoolsnn/blob/main/lauetoolsnn/example_notebook_scripts/Step2a_Optimize_architecture_LaueNN.ipynb)

Step 3: verify the neural network prediction and indexation of Laue Patterns: https://github.com/ravipurohit1991/lauetoolsnn/blob/main/lauetoolsnn/example_notebook_scripts/Step3_Prediction_LaueNN.ipynb

This script describes the steps for predicting Laue spots *hkl* for a given input and also includes functions that reconstructs orientation matrix and indexes the crystal of the given dataset. A script to generate simulated Laue patterns for prediction is also provided. (Step 3a: Generation of simulated dataset for prediction: https://github.com/ravipurohit1991/lauetoolsnn/blob/main/lauetoolsnn/example_notebook_scripts/Step3a_Generate_simulateLPforPrediction_LaueNN.ipynb)

The scripts have default values that should allow the user to launch all the scripts in sequence to see the working flow of the method described in this article.

2. Reproducibility of the data presented in article

The results of the indexation for the case studies presented in article can be found at https://github.com/ravipurohit1991/lauetoolsnn/tree/main/lauetoolsnn/example_case_study_paper. The trained model files are not uploaded due to size restriction but are available from the first author up on contact. Script is also provided to visualize the results.

3. Grid search optimization

Before the training, the problem of model overfitting should be analyzed as we don't have a balanced training dataset (due to crystal symmetry application). Some *hkl* reflection appear more often than others in the Laue pattern and this leads to an imbalance in the dataset. To address possible overfitting we have performed regularization of the neural network architecture. The grid search optimization of the hyper parameters of the multilayer perceptron (MLP) architecture is carried out with sklearn (https://scikit-learn.org/0.17/modules/generated/sklearn.grid_search.GridSearchCV.html) GridSearchCV algorithm. In the current model, a L2 norm (sum of squared weights) is applied to both kernel and bias parameters of each dense layers. This regularization seemingly improved the overfitting in the model. The regularization parameters were then searched over a regular grid of values from 0.1 to 1e-6. Figure 1 shows the training accuracy for FCC Cu plotted over the range of values during one epoch of training. This was done subsequently for all crystal symmetries. For FCC Cu, the optimized values of regularization were found to be (learning rate = 0.001, Bias coefficient = 1e-05, Kernel coefficient = 1e-06)

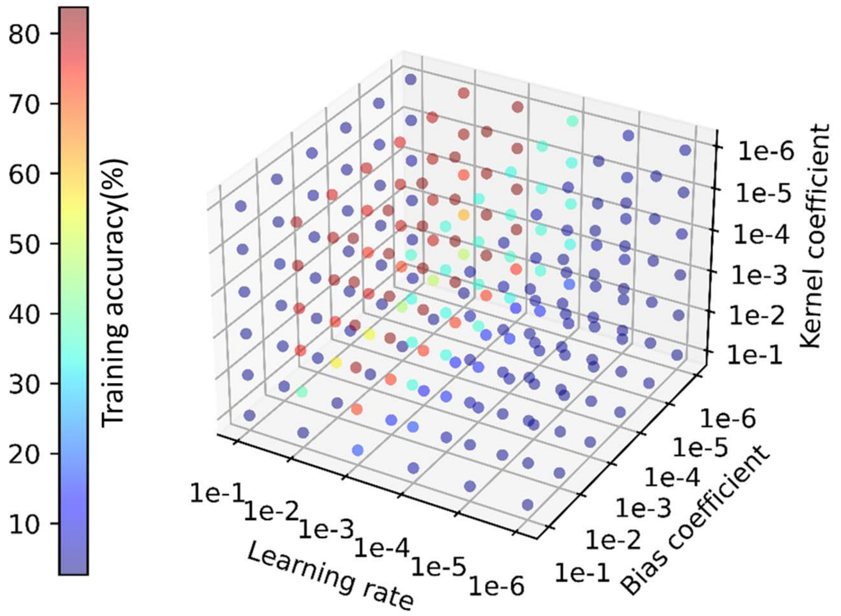

Figure 1 Grid optimization of learning rate of the optimizer along with the Kernal and Bias coefficients of the hidden layers in the neural network for one epoch.

Additional optimization of number of neurons per hidden layer, number of hidden layers, activation function, dropout ratio were optimized over different sets of materials of varied crystal symmetry and

their average values are taken for the optimized architecture presented in the article. A python notebook script is included in the lauetoolsnn repository (https://github.com/ravipurohit1991/lauetoolsnn/blob/main/lauetoolsnn/example_notebook_scripts/Step2a_Optimize_architecture_LaueNN.ipynb) which shows the strategy of grid search optimization used in this article.

4. Comparison of Classical indexation vs LaueNN

For comparison with classical indextion method, we have used Lauetools (https://gitlab.esrf.fr/micha/lauetools) software. There is no significant improvement of indexing when it comes to single crystal high crystal symmetry structures, except that the LaueNN method indexes faster a polycrystalline case than the conventional software. We indicate below the comparison in the case of low symmetry polycrystalline zirconia material.

A polycrystalline zirconia sample can produce more than 1000 Laue spots in the detector frame. A brute search approach using the conventional indexation with 8 CPUs take approximately 5 minutes (including peak search, orientation matrix construction and strain refinement) to index one crystal in such an image while the same process takes ~1s with LaueNN method. A flow chart of where the LaueNN method replaces the conventional indexation is presented in Figure 2 below.

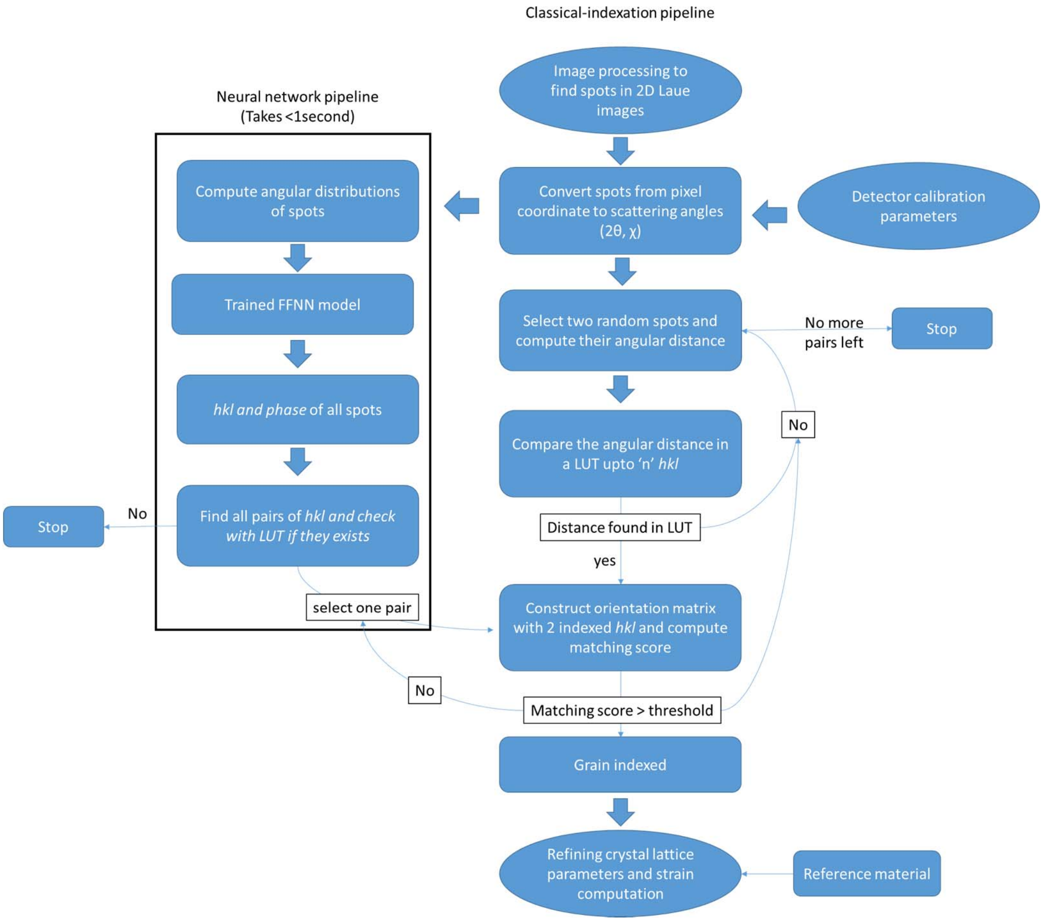

Figure 2 Laue pattern analysis workflow

5. Effect of training dataset on neural network training and validation

The training dataset here comprises of Laue patterns of uniform crystallographic orientation for a given symmetry. Figure 3 shows the uniform sampling of crystal orientation for copper (500 orientations), used to generate Laue patterns for the training. The figure on the right shows the 20% of training dataset used for validation. Care was to taken to avoid overlap between the training and validation dataset, as this could lead to strong overfitting. For high symmetry crystals often 500 orientations can sufficiently describe the orientation space uniformly whereas more orientations are required in case of low symmetry.

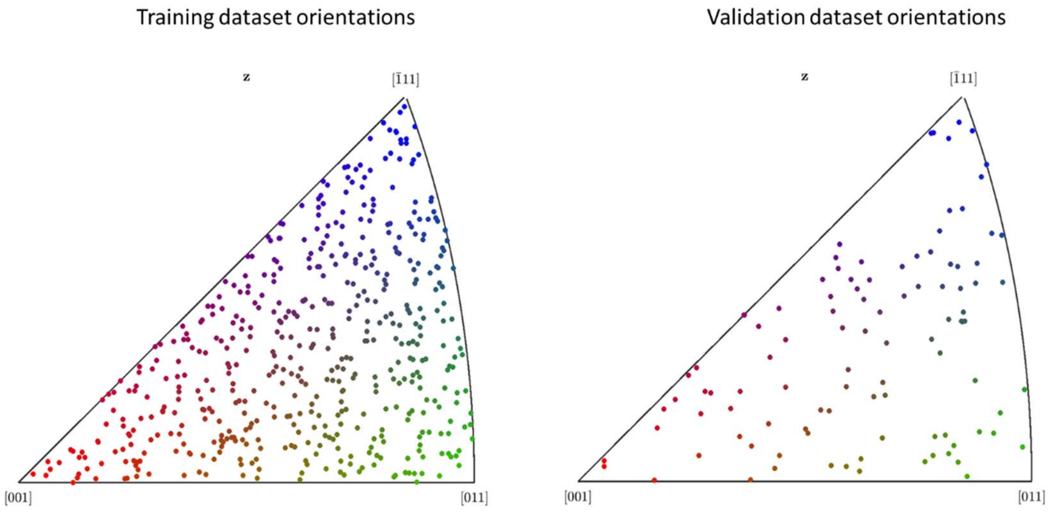

Figure 3 Inverse Pole Figure of the uniform distribution of orientations (cubic structure case) considered for (a) training dataset (80% of data) (b) validation dataset (20% of data). The orientations in validation dataset do not overlap with training dataset.

To analyze the effect of number of training dataset on the accuracy of MLP model, we performed training with varied number of training dataset. This is shown in the case of low-symmetry zirconia crystal in Figure 4. Similar to the strategy presented above for Cu crystal, the number of orientations that forms the training dataset is varied from 10 to 2000 and the neural network model is trained with 20 epochs. The performance of the model is then tested on randomly simulated polycrystalline (5 crystals) zirconia Laue patterns (on 100 multiple runs). As can be seen from Figure 4, for low symmetry crystal like zirconia at least 1000 orientations are required to build a training dataset to be able to achieve 90% validation accuracy.

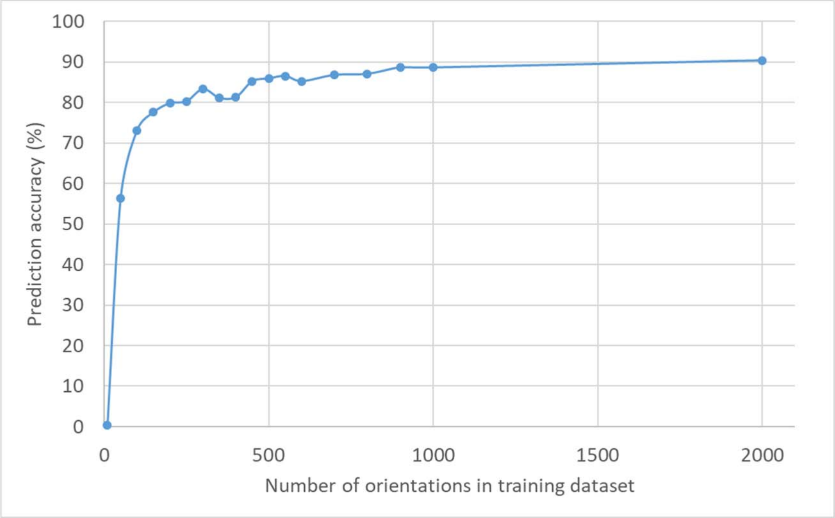

Figure 4 Effect of number of random orientations in training dataset to prediction accuracy of zirconia crystal.

Script detailing the effect of training dataset in the case of zirconia can be found at: https://github.com/ravipurohit1991/lauetoolsnn/blob/main/lauetoolsnn/example_notebook_scripts/Effect_of_training_dataset.ipynb
